# Supplementary material for: Dynamic diversity of synthetic supramolecular polymers in water as revealed by hydrogen/deuterium exchange
Source: Nat Commun. 2017 May 15;8:15420. doi: 10.1038/ncomms15420 (PMC5440672; doi:10.1038/ncomms15420)
Supplement: Supplementary Information — Supplementary Figures, Supplementary Tables, Supplementary Methods and Supplementary References [file ncomms15420-s1.pdf]

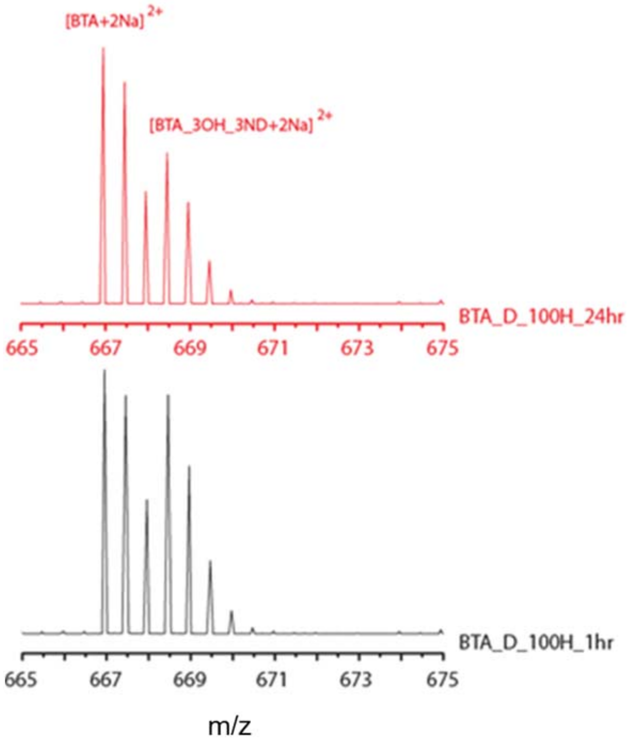

Supplementary Figure 1. HDX-MS spectra of C_{12}BTA fibers in the 'exchange-out' scenario after HDX for 1 hour (spectrum in black) and 24 hours (spectrum in red).

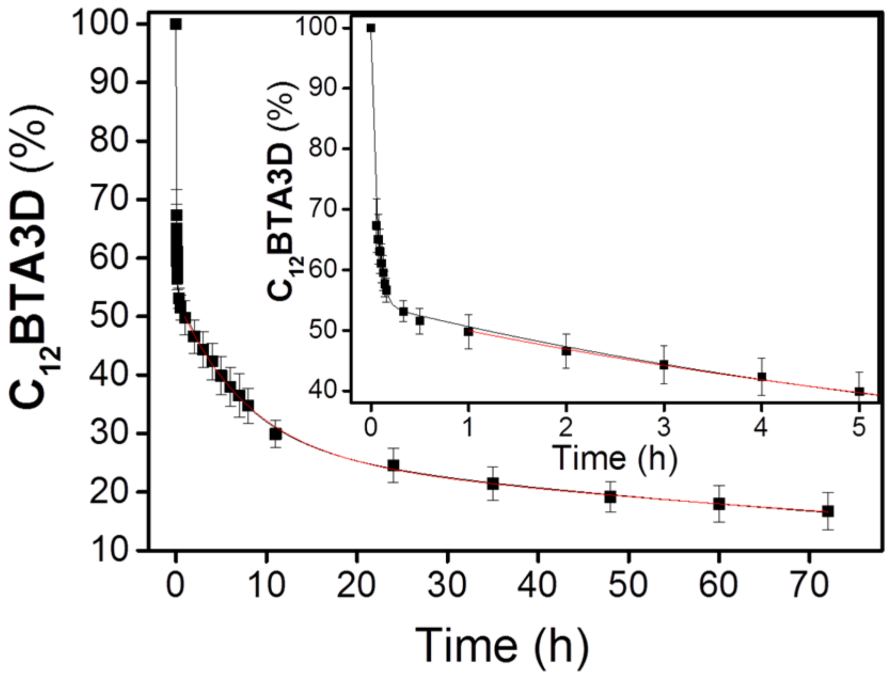

Supplementary Figure 2. Comparison of tri-exponential fit of the experimental data (percentage of C_{12}BTA3D as a function of time) including the point (t = 0 h, BTA3D = 100%) (black line), and bi-exponential fit of the experimental data starting from 1 h (red line). The inset shows an enlargement of the data points and fit for the first 5 hours. The error bars represent one standard deviation of uncertainty computed from three separate kinetic experiments. The rate constants for the tri-exponential fit are; k_{fast} = 1.4 ×10^{-1} h^{-1} and k_{slow} = 0.7 ×10^{-2} h^{-1}, and for the bi-exponential fit; k_{fast} = 1.3 ×10^{-1} h^{-1} and k_{slow} = 0.7 ×10^{-2} h^{-1}.

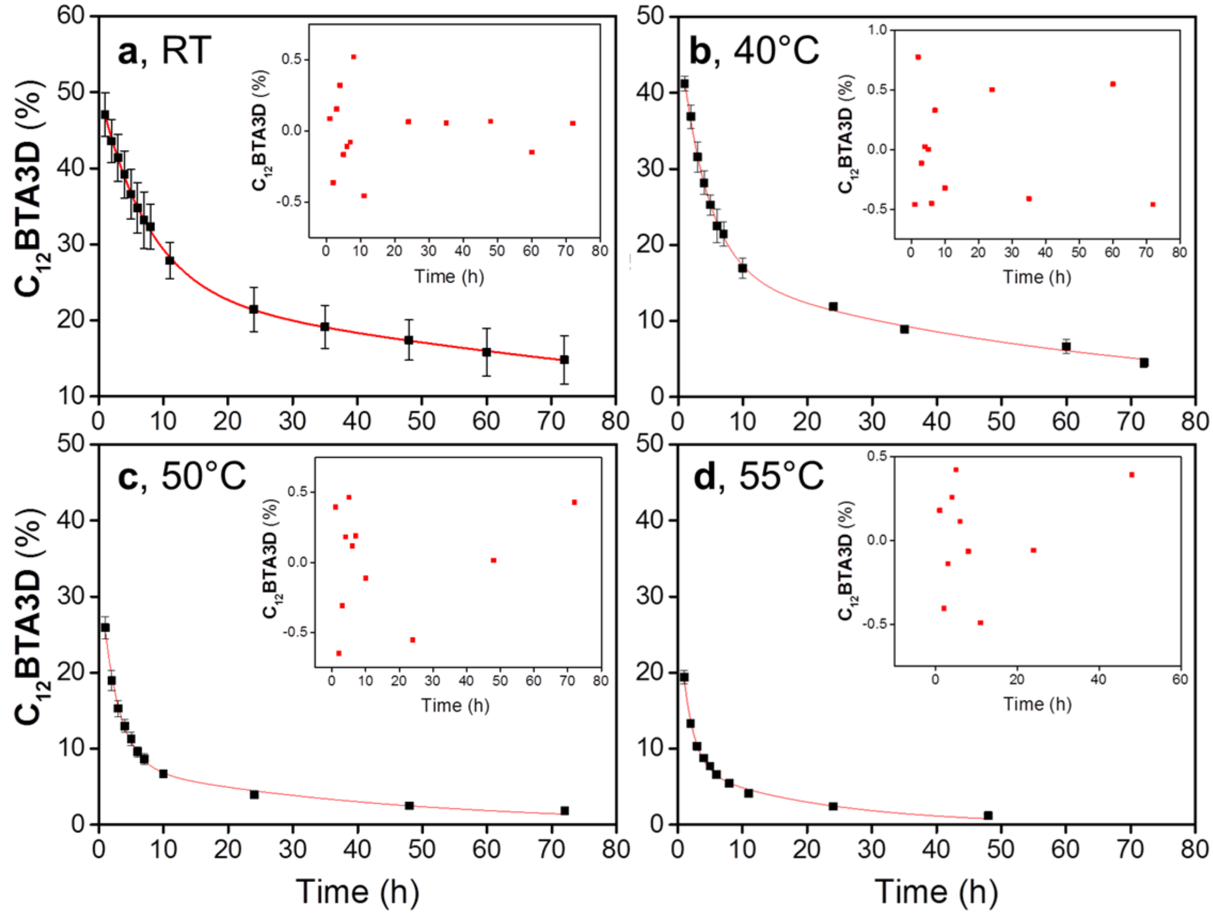

Supplementary Figure 3. Percentage of C_{12}BTA3D over time when equilibrated at temperatures of (**a**) room temperature, (**b**) 40°C, (**c**) 50°C and (**d**) 55°C, along with a plot of the residuals (insets). The error bars represent one standard deviation of uncertainty computed from three separate kinetic experiments. The rate constants are (**a**) k_{fast} = 1.3 ×10^{-1} h^{-1} and k_{slow} = 0.7 ×10^{-2} h^{-1}, (**b**) k_{fast} = 2.4×10^{-1} h^{-1} and k_{slow} = 1.7 ×10^{-2} h^{-1}, (**c**) k_{fast} = 3.9 ×10^{-1} h^{-1} and k_{slow} = 2.4 ×10^{-2} h^{-1} and (**d**) k_{fast} = 5.7 ×10^{-1} h^{-1} and k_{slow} = 4.8×10^{-2} h^{-1}.

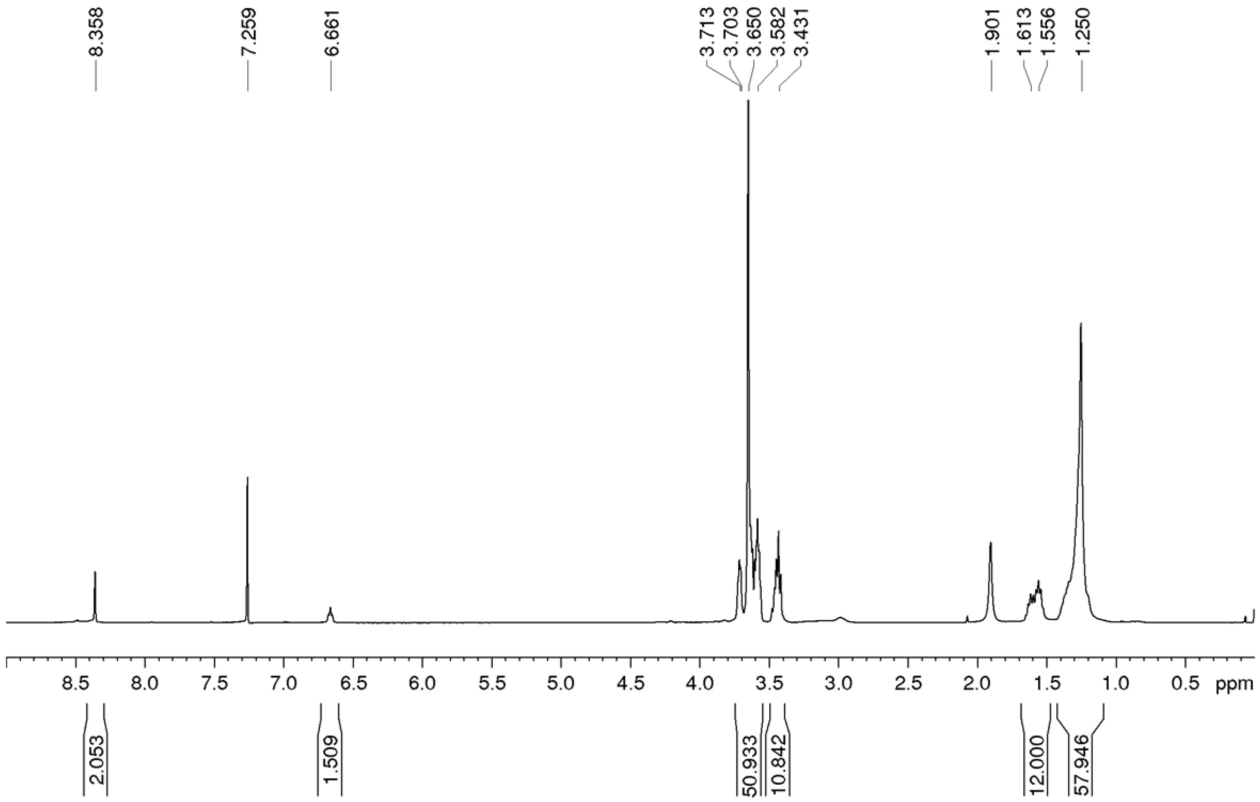

Supplementary Figure 4. ^{1}H NMR of $C_{13}BTA$ in $CDCl_3$.

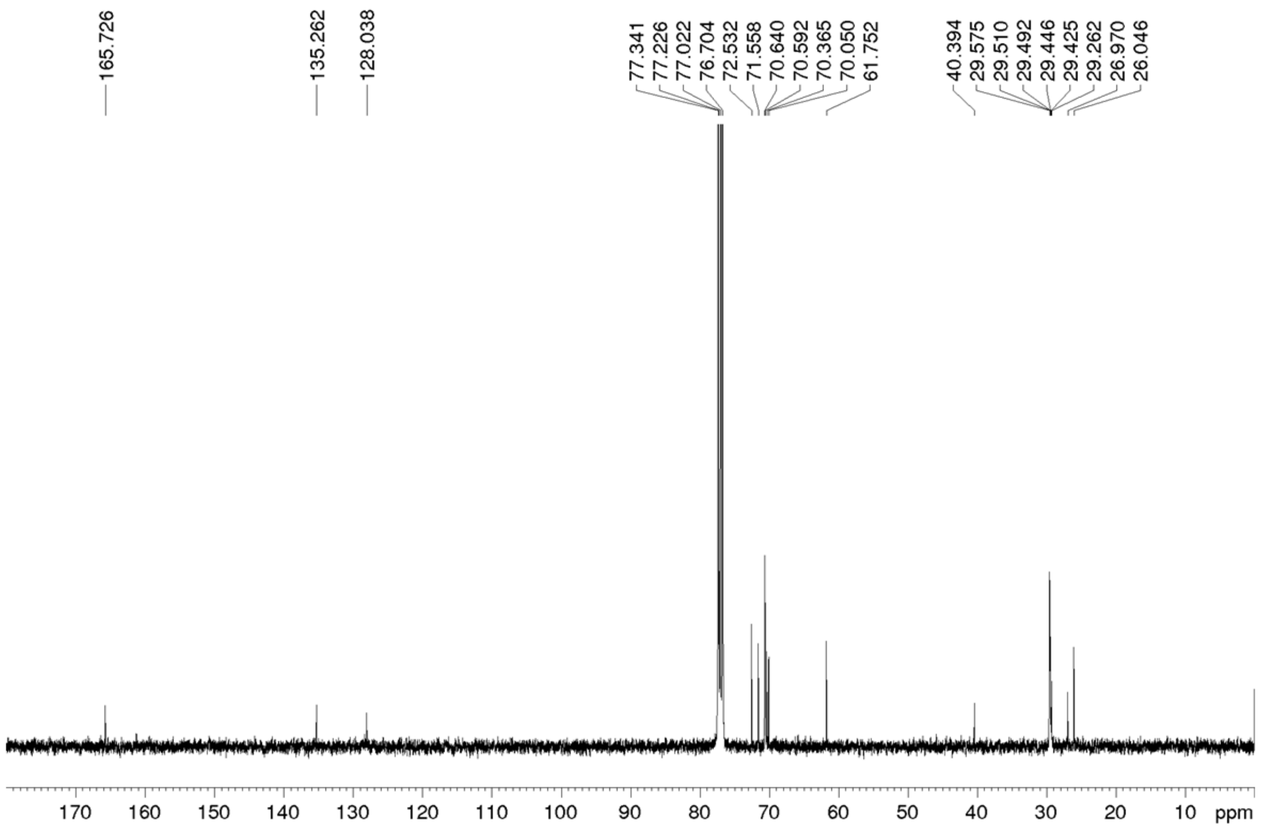

Supplementary Figure 5. ^{13}C NMR of C_{13}BTA in $CDCl_3$.

Comment 1 CHCA/DCTB in THF calibration in D:\Data\Users 2016\Ralf Bovee\April\Thuur_van_Onzen\April062016

Comment 2

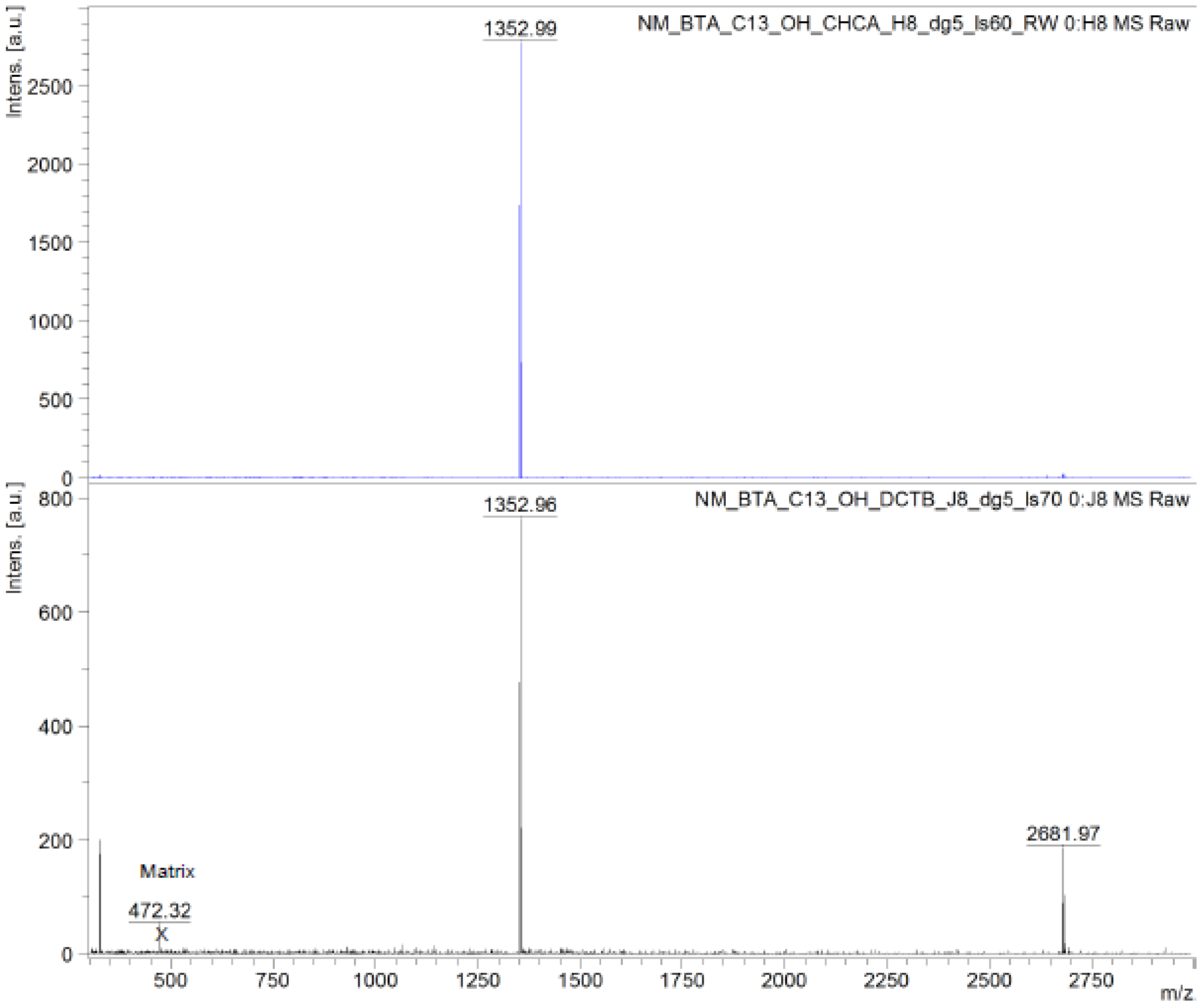

Acquisition Parameter

Date of acquisition	2016-04-06T15:22:50.227+02:00
Acquisition method name	D:\Data\Users 2016\Ralf Bovee\April\Thuur_van_Onzen\April062016\RP_700-3500_Da_06042016_serie1.par
Aquisition operation mode	Reflector
Voltage polarity	POS
Number of shots	500
Name of spectrum used for calibration	
Calibration reference list used	Cesium tri jodide positive mode

Instrument Info

User	smo379
Instrument	ST-A2130
Instrument type	autoflex

Supplementary Figure 6. MALDI-TOF-MS of C_{13}BTA.

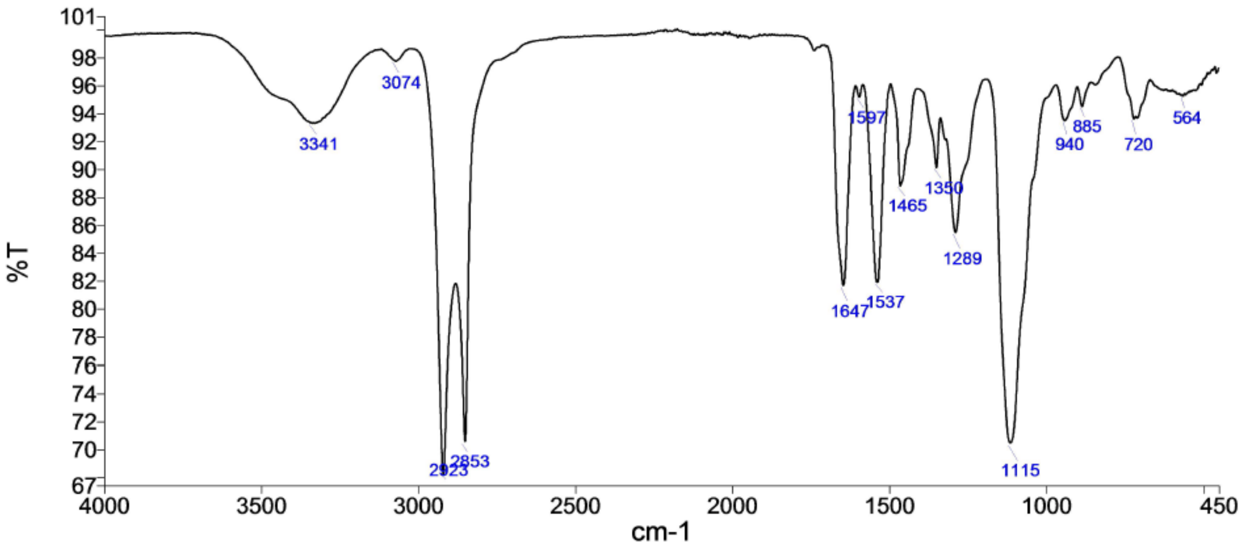

Supplementary Figure 7. IR spectrum of C_{13}BTA.

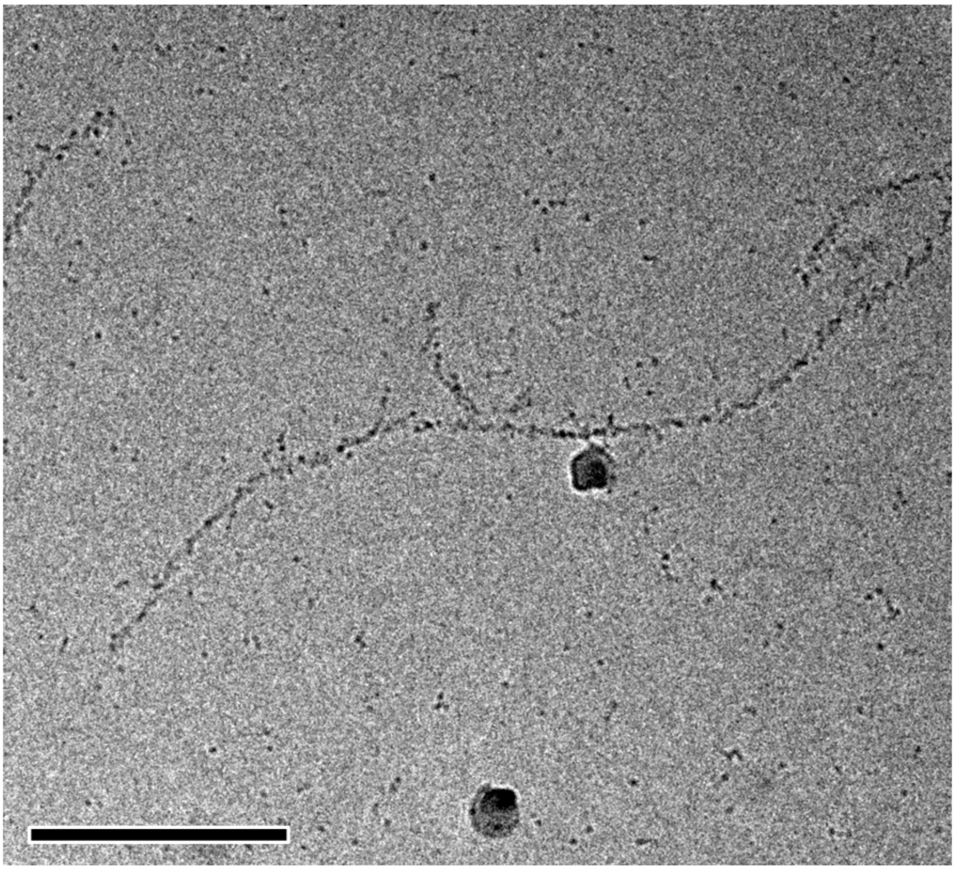

Supplementary Figure 8. Cryo-TEM image (scale bar 200 nm) of the C_{13}BTA at a concentration of 453 μM in H_2O, revealing the presence of supramolecular polymers coexisting with small spherical micelles at a magnification of 25000, 5 μm below focus. The two black sphere-like objects are crystalline ice particles.

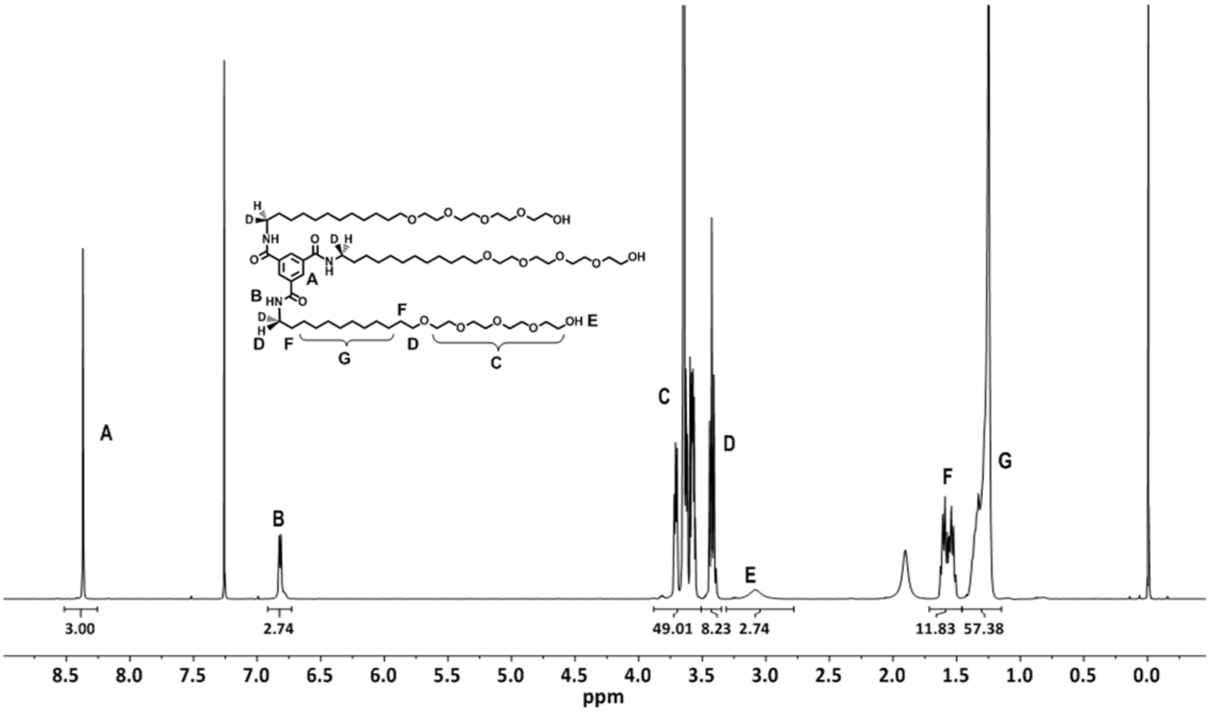

Supplementary Figure 9. ^{1}H NMR of (*S*)-D-C_{12}BTA in $CDCl_3$.

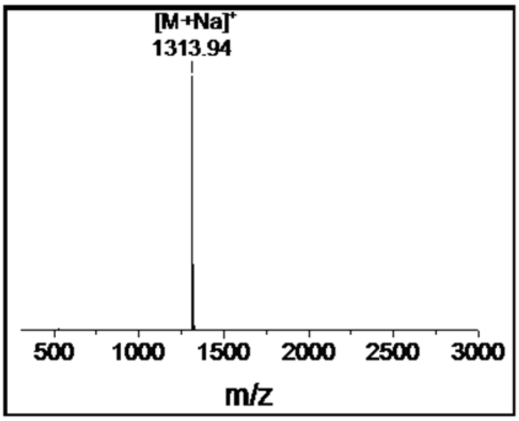

Supplementary Figure 10. MALDI-TOF-MS of (*S*)-D-C_{12}BTA.

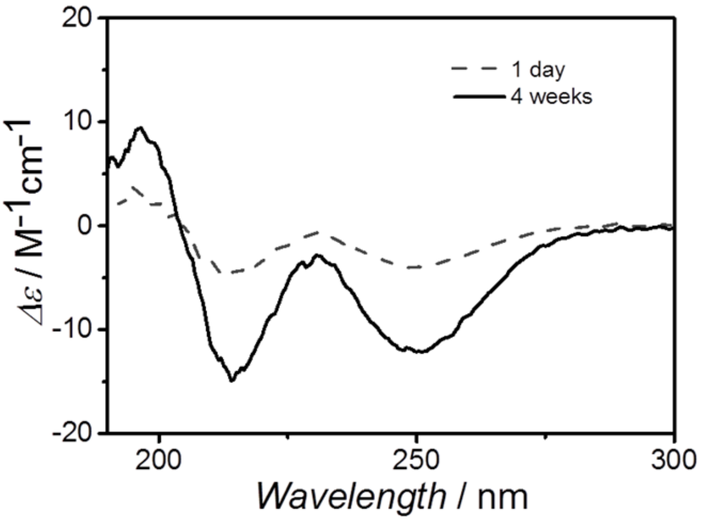

Supplementary Figure 11. CD spectra of (S)-D-C_{12}BTA in water after 1 day (dashed line), and after equilibration for 4 weeks (c = 50 μM, T = 20 °C, path length = 1 cm). The solution of (S)-D-C_{12}BTA was prepared by injection of a concentrated solution in methanol (5 μL, concentrations 15 mM and 25 mM) into H_2O (2.5 mL, final concentrations 30 μM and 50 μM), and the samples was equilibrated overnight.

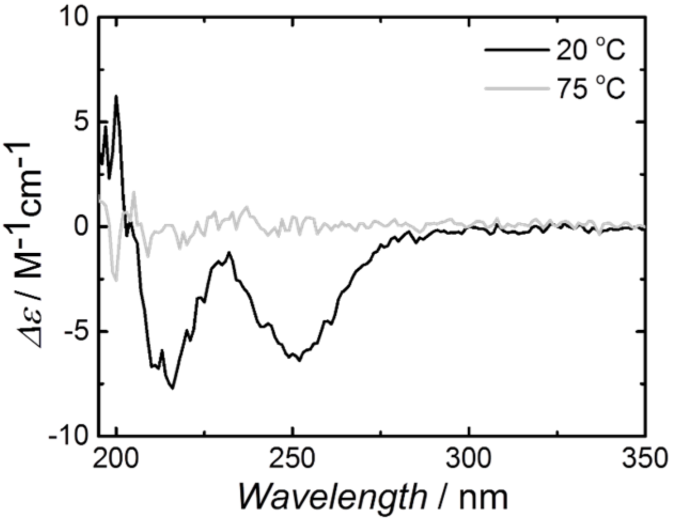

Supplementary Figure 12. CD spectrum of (S)-D-C_{12}BTA in water after 1 day (c = 30 μM) at two different temperatures. The solution of (S)-D-C_{12}BTA was prepared by injection of a concentrated solution in methanol (5 μL, concentrations 15 mM and 25 mM) into H_2O (2.5 mL, final concentrations 30 μM and 50 μM), and the samples was equilibrated overnight.

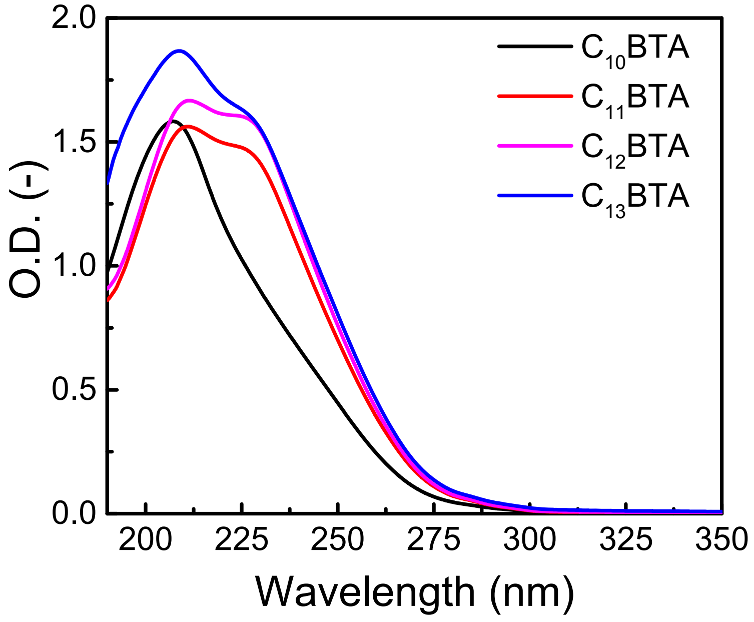

Supplementary Figure 13. UV-spectra of C_{10}BTA, C_{11}BTA, C_{12}BTA and C_{13}BTA at a concentration of 50 μM, measured at 20°C using a 1 cm path length quartz cuvet. As we previously reported, the C_{10}BTA displays a single maximum at 207 nm, whereas C_{11}BTA and C_{12}BTA display two absorption maxima at 211 and 226 nm[1]. C_{13}BTA has two absorption maxima at 209 and 226 nm; the relative absorption of the first peak as compared to the second peak is increased as compared to C_{11}BTA and C_{12}BTA.

Supplementary Table 1. Relative isotopic intensities of C_{12}BTA6D as compared to C_{12}BTA3D after HDX at 0°C for 1 minute and 1 hour .[a)]

	BTA/H_2O [e)]	HDX 1min	HDX 1 hour
$I_{668.46}$ [b)]	1.00	1.00	1.00
$I_{669.97}$ [c)]	0.10	0.17	0.86
C_{12}BTA6D% [d)]	0	6.5	43.2

a) A 500 μM solution of BTA/H_2O was diluted 100 times into D_2O; the BTA solution and D_2O were both cooled down to 0°C, just before the dilution.
b) $I_{668.46}$ is the base peak for doubly charged C_{12}BTA3D, and is arbitrarily set at 1.00.
c) $I_{669.97}$ is the total ion intensity at *m/z* of 669.97.
d) Calculated C_{12}BTA6D% based on the isotopic distribution by the equation C_{12}BTA6D% = ($I_{669.97}$ - 0.10)/[($I_{669.97}$ - 0.10) + 1].
e) Theoretical values of C_{12}BTA3D before H/D exchange (see also Supplementary Table 2).

Supplementary Table 2. Theoretical isotopic distributions of doubly charged sodiated C_{12}BTA, C_{12}BTA3D and C_{12}BTA6D ions. These calculations were performed using IsoPro Software. The isotopic distribution of $[C_{12}BTA + 2Na]^{2+}$ is displayed in Fig. 2b (the green spectrum).

C_{12}BTA $[(C_{69}H_{129}N_3O_{18}) + 2Na]^{2+}$		C_{12}BTA3D $[(C_{69}H_{126}N_3O_{18}D_3) + 2Na]^{2+}$		C_{12}BTA6D $[(C_{69}H_{123}N_3O_{18}D_6) + 2Na]^{2+}$	
Mass	abundance	mass	abundance	mass	abundance
666.95	100.00	668.46	100.00	**669.97**	**100.00**
667.45	77.86	668.96	77.86	670.47	77.86
667.96	33.60	669.46	33.60	670.97	33.60
668.46	10.43	**669.97**	**10.43**	671.47	10.43
668.96	2.58	670.47	2.58	671.98	2.58

Supplementary Table 3. Relative isotopic intensities of ions at *m/z* of 669.46 and at 669.97 as compared to C_{12}BTA3D (*m/z* at 668.46).[a)]

	0 hr [f)]	1 hr	24hr
$I_{668.46}$ [b)]	1.00	1.00	1.00
$I_{669.46}$ [c)]	0.34	0.42	0.59
$I_{669.97}$ [d)]	0.10	1.05	2.86
$cI_{669.46}$ [e)]	0.34	0.36	0.41

a) A 500 μM solution of BTA/H_2O was diluted 100 times into D_2O at room temperature.
b) $I_{668.46}$ is the base peak for doubly charged BTA3D, and is arbitrarily set at 1.00.
c) $I_{669.46}$ is the total ion intensity at *m/z* of 669.46.
d) $I_{669.97}$ is the total ion intensity at *m/z* of 669.97.
e) Corrected relative ion intensity at *m/z* of 669.46 by the equation of $cI_{669.46}$ = [$I_{669.46}$ -($I_{669.97}$ - 0.10) × 6.6%].
f) Theoretical values of BTA3D before H/D exchange.

Supplementary Table 4. C_{12}BTA3D% after HDX of BTA-based supramolecular polymers at different concentrations[a)].

HDX time	1 mM [b)]	0.5 mM [b)]	0.1 mM [b)]
5 min	58.5	57.1	58.4
30 min	47.8	47.5	45.5
1 hour	46.2	46.4	44.7
24 hour	36.0	34.9	36.3

a) The BTA samples prepared in H_2O were diluted 100 times into D_2O.
b) Concentrations of the BTA-H_2O solutions, all prepared according to the method described in the Methods section of the manuscript.

Supplementary Methods

1. Calculating the percentage of C_{12}BTA3D

Upon diluting a BTA-H_2O solution 100 times into D_2O, C_{12}BTA will quickly be transformed into C_{12}BTA3D, because the hydrogens of the OH groups of C_{12}BTA will be instantaneously replaced by deuterium. Subsequently, HDX of the amide groups will take place. When calculating the percentage of not exchanged C_{12}BTA, the overlapping isotopic peaks of C_{12}BTA3D and C_{12}BTA6D, and the presence of 1% H_2O (w/w, with molar ratio of 1.1%), should be taken into account. As can be seen in Table S2, the base peak of $[C_{12}BTA3D + 2Na]^{2+}$ (with a peak intensity of $I_{668.46}$) at *m/z* of 668.46 will result in an isotopic peak at *m/z* of 669.97 (with a peak intensity of 10.43% of $I_{668.46}$, in bold), that will overlap with the base peak of $[C_{12}BTA6D + 2Na]^{2+}$ (in bold). Considering the presence of 1.1% (molar ratio) of H_2O in a HDX solution, all hydrogen atoms (3 OH and 3 NH) cannot be completely replaced by deuterium. Statistically, the ratios of BTA4D : BTA5D : BTA6D are, 1.86×10^{-3} : 6.67×10^{-2} : 1. Although the amount of BTA4D formed is negligible, the amount of BTA5D (6.6%) has to be taken into consideration when calculating the percentage of C_{12}BTA3D. Based on the discussion above, BTA3D% is calculated by the following equation,

$$BTA3D\% = \frac{I_{668.46}}{I_{668.46} + ((I_{669.97} - 0.1043 I_{668.46}) \times 1.066)} \times 100$$

with $I_{668.46}$ and $I_{669.97}$ representing the intensity of the peaks for the doubly charged sodiated ions at *m/z* of 668.46 and 669.97, respectively.

As discussed above, the relative intensity of C_{12}BTA5D is influenced by the presence of the 1.1% (molar ratio) of H_2O. Hence, the theoretical ratio of BTA5D/BTA6D for the samples is 6.6%. The expected relative isotopic intensities of ions at *m/z* of 669.46 and at 669.97 as compared to C_{12}BTA3D (*m/z* at 668.46) of the BTA samples after HDX of 1 and 24 hours are given in Table S3. From Table S3, it can be seen that the corrected values of $cI_{669.46}$ are close to 0.34 but still slightly higher. The discrepancy might be due to the actual molar ratios of H_2O/D_2O is slightly higher than 1.1% because of the trace amount of H_2O in the hypothetically pure D_2O and the possible up-taking of small amount of H_2O from air by the sample in the long HDX process.

2. Synthesis of C_{13}BTA

The synthesis of the starting compound, 26-bromo-1-phenyl-2,5,8,11,14-pentaoxahexacosane, was reported previously[2].

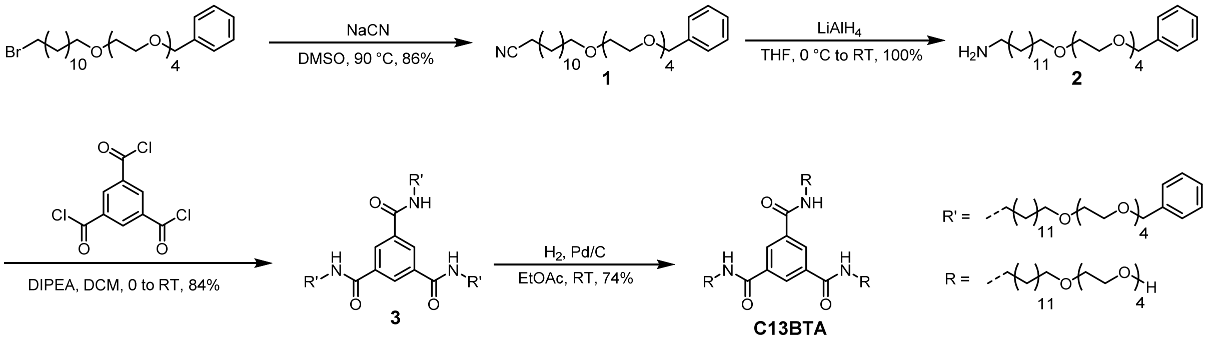

Supplementary Figure 14. Synthetic scheme for the synthesis of C_{13}BTA.

*1-Phenyl-2,5,8,11,14-pentaoxaheptacosane-27-nitrile (**1**).* A 12 ml vial equipped with a magnetic stirring bar was charged with sodium cyanide (0.277 g, 5.64 mmol), 26-bromo-1-phenyl-2,5,8,11,14-pentaoxahexacosane (2.0 g, 3.76 mmol), and DMSO (6 mL). The vial was sealed under an Ar atmosphere and stirred at 90 °C. After 3 h, the crude reaction mixture was diluted with diethyl ether (50 ml) and washed with water (4 x 50 ml). The organic layer was subsequently dried over $MgSO_4$, filtered, and concentrated *in vacuo* to yield the product, **1,** as a yellow oil (1.55 g, 86%). ^{1}H NMR (400 MHz, $CDCl_3$): δ = 7.38-7.23 (m, 5H), 4.56 (s, 2H), 3.70-3.60 (m, 14H), 3.44 (t, J = 6.81, 2H), 2.31 (t, J = 7.14, 2H), 1.70-1.51 (m, 4H), 1.49-1.38 (m, 2H), 1.36-1.21 (m, 14H). ^{13}C NMR (100 MHz, $CDCl_3$,): δ = 138.30, 128.33, 127.71, 127.55, 119.84, 73.21, 71.49, 70.64, 70.61, 70.01, 69.45, 29.63, 29.53, 29.50, 29.45, 29.27, 28.74, 28.64, 26.08, 25.35, 17.075. FTIR (ATR): v = 3031, 2924, 2854, 2245, 1496, 1454, 1351. MS (LC-ESI) calcd. for $C_{28}H_{47}NO_5$ $[M+H]^+$ 478.36; found: 478.42.

*1-Phenyl-2,5,8,11,14-pentaoxaheptacosan-27-amine (**2**).* An oven dried round bottom flask equipped with a magnetic stirring bar was charged with THF (50 mL) and cooled to 0 °C. $LiAlH_4$ (1 M in THF, 11 mL) was then added slowly using a syringe and allowed to stir at 0 °C. A solution of **1** (1.50 g, 3.76 mmol) in THF (10 mL) was then added dropwise to the stirring solution of $LiAlH_4$. The reaction mixture was warmed to room temperature and stirred overnight under an argon atmosphere. The reaction mixture was then cooled to 0 °C and diethyl ether was added (50 mL). The reaction mixture was then quenched by the stepwise addition of H_2O (0.42 mL), 15% aqueous NaOH (0.42 mL), and H_2O (1.25 mL). The reaction mixture was then warmed to room temperature and stirred for 30 minutes. The mixture was then dried over $MgSO_4$ and filtered. Solvent was subsequently removed under reduced pressure to afford **2** as a clear oil in quantitative yield. ^{1}H NMR (400 MHz, $CDCl_3$): δ = 7.36-7.23 (m, 5H), 4.57 (s, 2H), 3.70-3.59 (m, 14H), 3.58-3.53 (m, 2H), 3.43 (t, J = 6.73, 2H), 2.67 (t, J = 6.99), 1.68-1.56 (m, 6H), 1.35-1.17 (m, 18H). ^{13}C NMR (100 MHz, $CDCl_3$,): δ = 138.28, 128.36, 127.75, 127.59, 73.25, 71.56, 70.65, 70.62, 70.05, 69.44, 42.29, 33.90, 29.64l, 29.61, 29.52, 29.50, 26.91, 26.10. FTIR (ATR): v = 3380, 3030, 2922, 2853, 1604, 1496, 1454, 1351. MS (LC-ESI) calcd. for $C_{28}H_{51}NO_5$ $[M+H]^+$ 482.39; found: 482.67.

*N^1,N^3,N^5-tris(1-Phenyl-2,5,8,11,14-pentaoxaheptacosan-27-yl)benzene-1,3,5-tricarboxamide (**3**).* An oven dried round bottom flask equipped with a magnetic stirring bar was charged with **2** (1.0 g, 2.08 mmol), *N,N*-diisopropylethylamine (0.68 g, 5.27 mmol) and dry CH_2Cl_2 (18 mL). A solution of 1,3,5-benzenetricarbonyl trichloride (0.17 g, 0.64 mmol) in dry CH_2Cl_2 (2 mL) was prepared in a separate vessel then added dropwise using a syringe to the solution containing **2**. The reaction mixture was stirred for 20 minutes at 0 °C and then allowed to warm to room temperature and stirred overnight in under an argon atmosphere. CH_2Cl_2 was then removed under reduced pressure and the resulting crude product was purified by column chromatography (silica gel, gradient from 0 to 5% MeOH in $CHCl_3$) to yield **3** as a clear viscous oil (0.86 g, 84%). ^{1}H NMR (400 MHz, $CDCl_3$): δ = 8.39 (m, 3H), 7.38-7.21 (m, 5H), 4.55 (s, 6H), 3.74-3.52 (m, 48H), 3.49-3.32 (m, 12H), 1.70-1.47 (m, 12H), 1.44-1.04 (m, 54H). ^{13}C NMR (100 MHz, $CDCl_3$,): δ = 165.71, 138.20, 135.26, 128.33, 127.72, 73.22, 71.53, 70.62, 70.61, 70.59, 70.02, 69.41, 40.23, 29.59, 29.55, 29.52, 29.48, 29.30, 27.01, 26.07, 26.05. FTIR (ATR): ν = 3249, 3064, 2923, 2853, 1641, 1541, 1496, 1454. MS (MALDI-TOF) calcd. for $C_{93}H_{153}N_3O_{18}$ $[M+Na]^+$ 1623.10; found: 1623.10.

N^1,N^3,N^5-tris(1-Hydroxy-3,6,9,12-tetraoxapentacosan-25-yl)benzene-1,3,5-tricarboxamide (C_{13}BTA). A Parr shaker type hydrogenation apparatus bottle was charged with **3** (0.82 g, 0.51 mmol) and ethyl acetate (30 ml). The mixture was degassed by sparging with nitrogen gas for 5 minutes. A catalytic amount of 10 wt% Pd/C was added and the reaction mixture was sparged with nitrogen gas for an additional 5 minutes. Hydrogenation was carried out overnight at room temperature on a Parr shaker type hydrogenation apparatus at 75 psi. The crude reaction mixture was then filtered through celite and the solvent was removed *in vacuo*. The crude product was subsequently purified via column chromatography (silica gel, gradient from 0 to 10% MeOH in $CHCl_3$) to yield C_{13}BTA as a colorless solid (0.50 g, 74%). ^{1}H NMR (400 MHz, $CDCl_3$): δ = 8.36 (s, 3H), 6.71-6.62 (m, 3H), 3.77-3.53 (m, 48H), 3.51-3.37 (m, 12H), 1.69-1.49 (m, 12H), 1.44-1.12 (m, 54H). ^{13}C NMR (100 MHz, $CDCl_3$,): δ = 165.76, 135.25, 128.05, 72.63, 71.57, 70.61, 70.57, 70.54, 70.29, 70.03, 61.72, 40.40, 29.53, 29.45, 29.42, 29.26, 26.97, 26.03. FTIR (ATR): ν = 3341 (broad), 3074, 2923, 2853, 1647, 1597, 1537, 1465. MS (MALDI-TOF) calcd. for $C_{72}H_{135}N_3O_{18}$ $[M+Na]^+$ 1352.96; found: 1352.96.

3. Cryo-TEM imaging of C_{13}BTA

Cryogenic transmission electron microscopy was performed using a C_{13}BTA sample at a concentration of 453 μM. Vitrified films were prepared in a 'Vitrobot' instrument (PC controlled vitrification robot, patent applied, Frederik et al 2002, patent licensed to FEI) at 22°C and at a relative humidity of 100%. In the preparation chamber of the 'Vitrobot', a 3 μL sample was applied on a Quantifoil grid (R 2/2, Quantifoil Micro Tools GmbH), which was surface plasma treated just prior to use (Cressington 208 carbon coater operating at 5 mA for 40 s). Excess sample was removed by blotting using filter paper for 3 s at –3 mm, and the thin film thus formed was plunged

(acceleration about 3 g) into liquid ethane just above its freezing point. The vitrified film was transferred to a cryoholder (Gatan 626) and observed at temperatures below -170 °C in a Tecnai Sphera microscope operating at 200 kV. Micrographs were taken at low dose conditions, with typical defocus settings of 5 and 10 µm at a magnification of 25000.

4. HDX kinetic fits for C_{11}BTA, C_{12}BTA and C_{13}BTA

The tri-exponential fits as displayed in Figure 5 were obtained as described in the Methods section of the manuscript. The equations representing the data are, for C_{11}BTA: $y(t) = 54.80 \times \exp(-t \times 33.27) + 8.12 \times \exp(-t \times 0.57) + 36.97 \times \exp(-t \times 0.015)$, for C_{12}BTA: $y(t) = 44.19 \times \exp(-t \times 17.57) + 9.57 \times \exp(-t \times 0.92) + 45.87 \times \exp(-t \times 0.009)$ and for C_{13}BTA: $y(t) = 21.77 \times \exp(-t \times 30.94) + 27.36 \times \exp(-t \times 1.57) + 50.82 \times \exp(-t \times 0.003)$. The adjusted R^2 of all fits was above 0.99.

5. Synthesis of (*S*)-D-C_{12}BTA

We prepared (*S*)-D-C_{12}BTA by introducing a deuterium isotope stereoselectively at the α-position of all dodecyl spacers (Supplementary Scheme 2)[3,4]. The enantioselective reduction in the third step was performed with the alcohol dehydrogenase of *Thermoanaerobacter sp.* (ADH-T) and isopropanol-d_8 as deuterium source as previously reported[5]. Phosphate buffered saline and isopropanol-*d8* were obtained from Sigma-Aldrich. Cofactor NADPH and alcohol dehydrogenase from *Thermoanaerobacter sp.* (ADH-T, (*S*)-selective, 331 U/mL) were purchased from Julich Chiral Solutions GmbH.

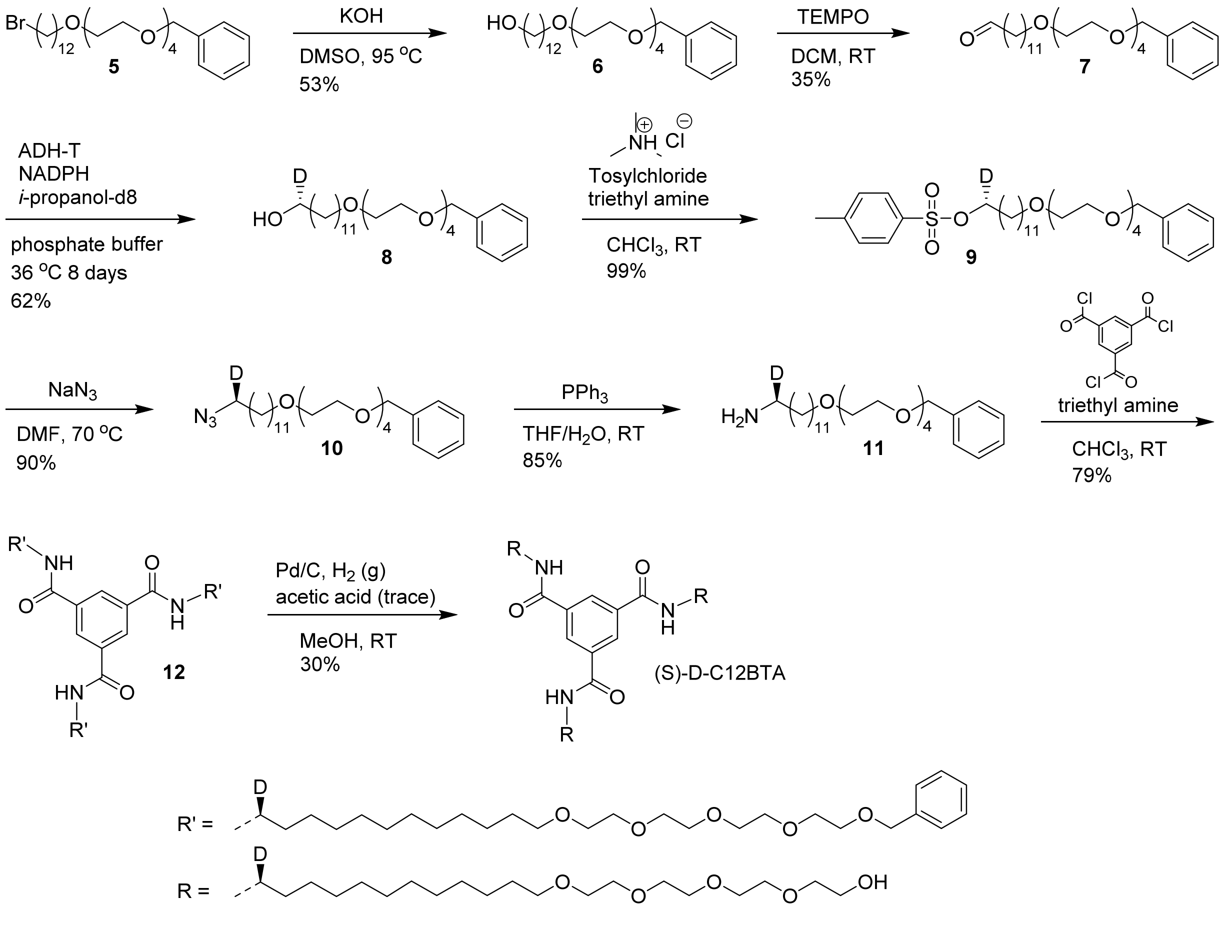

Supplementary Figure 15. Synthetic scheme for the synthesis of (*S*)-D-C_{12}BTA.

1-Phenyl-2,5,8,11,14-pentaoxahexacosan-26-ol (**6**).

A round bottom flask (50 mL) was charged with 26-bromo-1-phenyl-2,5,8,11,14-pentaoxahexacosane **5** (1.88 mmol, 1.0 g) and DMSO (5mL). To the stirring solution was added KOH (3.6 mmol, 0.2 g, powder) upon which the reaction mixture turned black. The reaction mixture was stirred overnight at 95 °C. The reaction mixture was concentrated *in vacuo*, redissolved in DCM (30 mL) and extracted with H_2O (3x 30 mL) and brine (2x 30 mL). The organic fraction was dried with $MgSO_4$, filtered and concentrated. The resulting material was purified by column chromatography (heptane/ethylacetate 40/60 v/v) yielding the target product with a small amount of dimer as a result of ether formation, which was removed in the next step (0.47 g, 53%). ^{1}H NMR (400 MHz, $CDCl_3$ δ): 7.42 – 7.28 (m, 5H, Ar), 4.57 (s, 2H, Ar-C$\underline{H}_2$-O), 3.71 – 3.60 (m, 16H, O-(C$\underline{H}_2$)$_2$-O, CH_2C$\underline{H}_2$-OH), 3.60 – 3.54 (m, 2H, O-(C$\underline{H}_2$)$_2$-O), 3.44 (t, *J* = 6.8 Hz, 2H, CH_2CH_2C$\underline{H}_2$$OCH_2$), 3.38 (t, *J* = 6.8 Hz, 0.3H, CH_2C$\underline{H}_2$$OCH_2$ CH_2, ether dimer side product), 1.64 – 1.46 (m, 4H, CH_2C$\underline{H}_2$$CH_2O$, C$\underline{H}_2$$CH_2Br$), 1.45 – 1.17 (m, 16H, aliphatic).

1-Phenyl-2,5,8,11,14-pentaoxahexacosan-26-al (**7**).

In a two neck flask (10 mL) 1-phenyl-2,5,8,11,14-pentaoxahexacosan-26-ol **6** (0.63 mmol, 0.297 g) was dissolved in dry DCM (3.5 mL) under an atmosphere of dry argon, and trichloroisocyanuric acid (0.7 mmol, 0.162 g) was added to the solution while stirring. Subsequently, the reaction mixture was placed in an ice bath and TEMPO (catalytic amount) was added, and the mixture was stirred for 10 minutes after which the ice bath was removed. The reaction mixture was stirred for an additional 40 minutes, after which it was filtered over celite and the reaction mixture was concentrated *in vacuo*. The solution was extracted with Na_2CO_3 (20 mL, saturated), HCl (20 mL, 1 M) and brine (20 mL). The organic phase was dried with $MgSO_4$, filtered and concentrated *in vacuo*. The resulting material was purified by column chromatography (heptane/ethyl acetate 40/60 v/v) yielding the target compound as a colourless oil (0.10 g, 35%). ^{1}H NMR (400 MHz, $CDCl_3$ δ): 9.76 (t, *J* = 1.9 Hz, 1H, O=C<u>H</u>CH$_2$), 7.48 – 7.27 (m, 5H, Ar), 4.57 (s, 2H, Ar-C<u>H</u>$_2$-O), 3.74 – 3.60 (m, 14H, O-(C<u>H</u>$_2$)$_2$-O), 3.61 – 3.52 (m, 2H, O-(C<u>H</u>$_2$)$_2$-O), 3.44 (t, *J* = 6.8 Hz, 2H, CH$_2$CH$_2$C<u>H</u>$_2$OCH$_2$), 2.41 (td, *J* = 7.3, 1.9 Hz, 2H, O=CHC<u>H</u>$_2$), 1.67 – 1.49 (m, 4H,), 1.28 (m, 16H, aliphatic). LC-ESI-MS: calculated M_w = 466.33 g/mol, observed m/z = 467.17 [H^+ adduct].

(R)-26-Deuterium-1-phenyl-2,5,8,11,14-pentaoxahexacosan-26-ol (**8**).

A round bottom flask (25 mL) was charged with 1-phenyl-2,5,8,11,14-pentaoxahexacosan-26-al **7** (0.21 mmol, 0.096 g), *i*-propanol-d8 (0.5 mL) and phosphate buffer (1.8 mL). To the stirring reaction mixture NADPH was added (13 mg) and mixture was heated to 36 °C. The enzyme ADH-T (0.01 mL) was added and the reaction mixture was stirred for 8 days. Subsequently, the aqueous reaction mixture was extracted with chloroform (3x 10 mL), the organic fractions were combined, dried with $MgSO_4$, filtered and concentrated *in vacuo*. The resulting material was purified by reversed phase column chromatography (acetonitrile/water 80/20 v/v) yielding the target compound in good purity (0.060 g, 62%). ^{1}H NMR (400 MHz, $CDCl_3$ δ): 7.41 – 7.27 (m, 5H, Ar), 4.57 (s, 2H, Ar-C<u>H</u>$_2$-O), 3.77 – 3.60 (m, 15H, O-(C<u>H</u>$_2$)$_2$-O, CH$_2$C<u>H</u>D-OH), 3.60 – 3.53 (m, 2H, O-(C<u>H</u>$_2$)$_2$-O), 3.44 (t, *J* = 6.8 Hz, 2H, CH$_2$CH$_2$C<u>H</u>$_2$OCH$_2$), 1.79 – 1.46 (m, 4H, CH$_2$C<u>H</u>$_2$CH$_2$O, C<u>H</u>$_2$CHD-OH), 1.47 – 1.09 (m, 16H, aliphatic). LC-ESI-MS: calculated M_w = 469.35 g/mol, observed m/z = 470.42 [H^+ adduct].

(R)-26-Deuterium-1-phenyl-2,5,8,11,14-pentaoxahexacosan-26-yl tosylate (**9**).

In a round bottom flask (10 mL) (*R*)-26-deuterium-1-phenyl-2,5,8,11,14-pentaoxahexacosan-26-ol **8** (0.13 mmol, 61 mg) was dissolved in dry chloroform (1 mL) under an atmosphere of dry argon and triethyl amine (0.05 mL) and trimethyl ammonium chloride (0.03 mmol, 2.6 mg) were added. The reaction mixture was

placed in an icebath and tosyl chloride (0.26 mmol, 51 mg) was slowly added. The ice bath was removed after 15 minutes and the reaction mixture was stirred overnight. Subsequently, the reaction mixture was diluted with chloroform (9 mL) and extracted with water (3x 10 mL) and brine (10 mL). The organic fraction was dried with MgSO4, filtered and concentrated in vacuo yielding the product as a white solid (81 mg, quantitative). ^{1}H NMR (400 MHz, $CDCl_3$ δ): 7.98 – 7.69 (m, 2H, Ar, tosyl), 7.51 – 7.28 (m, 7H, Ar, tosyl, benzyl), 4.57 (s, 2H, Ar-C<u>H</u>$_2$-O), 4.00 (t, *J* = 6.6 Hz, 1H, S-O-C<u>H</u>DCH_2), 3.79 – 3.60 (m, 14H, O-(C<u>H</u>$_2$)$_2$-O), 3.57 (m, 2H, O-(C<u>H</u>$_2$)$_2$-O), 3.44 (t, *J* = 6.8 Hz, 2H, CH_2CH_2C<u>H</u>$_2$OCH_2), 2.45 (s, 3H, C<u>H</u>$_3$-tosyl), 1.78 – 1.49 (m, 4H, CH_2C<u>H</u>$_2$$CH_2$O, C<u>H</u>$_2$CHD-OH), 1.49 – 1.10 (m, 16H, aliphatic). LC-ESI-MS: calculated M_w = 623.36 g/mol, observed m/z = 624.33 [H^+ adduct].

(S)-26-Deuterium-1-phenyl-2,5,8,11,14-pentaoxahexacosan-26-yl azide (**10**).

In a round bottom flask (5 mL) (R)-26-deuterium-1-phenyl-2,5,8,11,14-pentaoxahexacosan-26-yl tosylate **9** was dissolved in dry DMF (1 mL) and sodium azide (0.4 mmol, 28 mg) was added under an atmosphere of dry argon. The reaction mixture was heated to 70 °C and stirred overnight. Subsequently, brine was added (10 mL) and the reaction mixture was extracted with chloroform (3x 10 mL). The organic phases were combined, dried with $MgSO_4$, filtered and concentrated *in vacuo*. Remaining DMF was removed by a stream of nitrogen overnight yielding the product as a yellow oil (57.5 mg, 90%).). ^{1}H NMR (400 MHz, $CDCl_3$ δ): 7.38 – 7.27 (m, 5H, Ar), 4.57 (s, 2H, Ar-C<u>H</u>$_2$-O), 3.73 – 3.60 (m, 14H, O-(C<u>H</u>$_2$)$_2$-O), 3.60 – 3.53 (m, 2H, O-(C<u>H</u>$_2$)$_2$-O), 3.44 (t, *J* = 6.8 Hz, 2H, CH_2CH_2C<u>H</u>$_2$OCH_2), 3.24 (tt, *J* = 7.1, 1.8 Hz, 1H, N_3C<u>H</u>DCH_2), 1.74 – 1.49 (m, 4H, CH_2C<u>H</u>$_2$$CH_2$O, C<u>H</u>$_2$CHD-$N_3$), 1.46 – 1.13 (m, 16H, aliphatic). ^{13}H NMR (100 MHz, $CDCl_3$ δ): 138.25, 128.32, 127.70, 127.55, 73.22, 71.52, 70.63, 70.63, 70.60, 70.60, 70.59, 70.58, 70.03, 69.42, 51.13 (t), 29.62, 29.55, 29.53, 29.49, 29.46, 29.44, 29.13, 28.71, 26.66, 26.07. FT-IR (ATR) v (cm^{-1}): 3031, 2926, 2855, 2093, 1496, 1455, 1350, 1323, 1282, 1253, 1107, 1043, 1029, 992, 949, 880, 849, 818, 737, 723, 698, 647, 615, 551. LC-ESI-MS: calculated M_w = 494.36 g/mol, observed m/z = 517.50 [Na^+ adduct].

(S)-26-Deuterium-1-phenyl-2,5,8,11,14-pentaoxahexacosan-26-yl amine (**11**).

A round bottom flask (5 mL) was charged with (*R*)-26-deuterium-1-phenyl-2,5,8,11,14-pentaoxahexacosan-26-yl azide **10** (0.12 mmol, 57.5 mg) and THF (1 mL) and water (catalytic amount) were added. Triphenyl phosphine (0.35 mmol, 92 mg) was added to the reaction mixture and the clear solution was stirred overnight. The solvent was removed *in vacuo* and the resulting material was purified by silica filtration (chloroform/methanol 95/5, chloroform/methanol/isopropyl amine 85/5/10 v/v) yielding **11** as a slightly yellow oil which solidified upon standing (46 mg, 85%). ^{1}H NMR (400 MHz, $CDCl_3$ δ): 7.39 – 7.26 (m, 5H, Ar), 4.55 (s, 2H, Ar-C<u>H</u>$_2$-O), 3.70 –

3.59 (m, 14H, O-($C\underline{H}_2$)$_2$-O), 3.58 – 3.51 (m, 2H, O-($C\underline{H}_2$)$_2$-O), 3.42 (t, *J* = 6.8 Hz, 2H, $CH_2CH_2C\underline{H}_2OCH_2$), 2.65 (t, *J* = 7.2 Hz, 1H, $CH_2C\underline{H}D$-NH_2), 1.55 (p, *J* = 7.0 Hz, 2H, $CH_2C\underline{H}_2CH_2O$), 1.42 (p, *J* = 6.8 Hz, 2H, $C\underline{H}_2CHD$-NH_2), 1.35 – 1.13 (m, 16H, aliphatic). ^{13}H NMR (100 MHz, $CDCl_3$ δ): 138.25, 128.32, 127.71, 127.54, 73.21, 71.52, 70.63, 70.62, 70.61, 70.59, 70.58, 70.57 70.03, 69.41, 41.93 (t), 33.39, 29.62, 29.59, 29.56, 29.56, 29.46, 29.46, 26.83, 26.07. FT-IR (ATR) v (cm^{-1}): 30.30, 2924, 2855, 1686, 1638, 1567, 1465, 1455, 1351, 1300, 1250, 1202, 1175, 1111, 1043, 992, 945, 878, 831, 800, 737, 721, 698, 545. LC-ESI-MS: calculated M_w = 468.37 g/mol, observed m/z = 469.58 [H^+ adduct].

N^1,N^3,N^5-tris((S)-26-Deuterium-1-phenyl-2,5,8,11,14-pentaoxahexacos-20-en-26-yl)benzene-1,3,5-tricarboxamide (**12**).

A two neck flask (10 mL) was charged with (*S*)-26-deuterium-1-phenyl-2,5,8,11,14-pentaoxahexacosan-26-yl amine **11** and dry chloroform (0.5 mL) and triethyl amine (0.02 mL) were added under an atmosphere of dry argon. The reaction mixture was placed in an ice bath and a solution of 1,3,5-benzenetricarbonyl trichloride (0.03 mmol, 7.73 mg) in dry chloroform (0.5 mL) was added dropwise to the stirring solution. The ice bath was removed after 15 minutes and the reaction mixture was stirred at room temperature overnight. Subsequently, the solvent was removed *in vacuo* and the resulting material was purified by column chromatography (heptane/dimethoxyethane, 1/1, v/v) yielding **12** as an off white solid (36 mg, 79%). ^{1}H NMR (400 MHz, $CDCl_3$ δ): 8.39 (s, 3H, Ar, benzenetricarboxamide), 7.40 – 7.28 (m, 15H, Ar, benzyl), 6.77 (d, J = 5.7 Hz, 3H, C=ON$\underline{H}$CHDCH_2), 4.54 (s, 6H, Ar-$C\underline{H}_2$-O), 3.76 – 3.50 (m, 51H, O-($C\underline{H}_2$)$_2$-O, C=ONHC$\underline{H}$DCH_2), 3.42 (t, J = 6.8 Hz, 6H, $CH_2CH_2C\underline{H}_2O$), 1.67 – 1.46 (m, 12H, $CH_2C\underline{H}_2CH_2O$, $C\underline{H}_2$CHD-NHC=O), 1.45 – 1.16 (m, 48H, aliphatic). ^{13}H NMR (100 MHz, $CDCl_3$ δ): 165.75, 138.16, 135.23, 128.31, 128.11, 127.72, 127.56, 73.22, 71.51, 70.64-70.53, 70.01, 69.40, 40.03 (t), 29.57, 29.50, 29.48-29.42, 29.40, 29.25, 26.93, 26.03. FT-IR (ATR) v (cm^{-1}): 3240, 3064, 3034, 2924, 2854, 1640, 1555, 1496, 1454, 1351, 1298, 1257, 1107, 1029, 993, 946, 880, 846, 736, 697, 616, 472. MALDI-ToF-MS: calculated M_w = 1561.09 g/mol, observed m/z = 1584.07 [Na^+ adduct].

N^1,N^3,N^5-Tris((S)-1-hydroxy-24-deuterium-3,6,9,12-tetraoxatetracosan-24-yl)benzene-1,3,5-tricarboxamide ((S)-D-C_{12}BTA).

A round bottom flask(25 mL) was charged with *N*^{1},*N*^{3},*N*^{5}-tris((*S*)-26-deuterium-1-phenyl-2,5,8,11,14-pentaoxahexacos-20-en-26-yl)benzene-1,3,5-tricarboxamide **12** (0.023 mmol, 36 mg) and methanol was added (1 mL). After N_2(g) was led through the stirred solution for 10 minutes, Pd/C (catalytic amount) was added and a balloon filled with H_2(g) was connected. Because the reaction proceeded poorly, acetic acid (catalytic amount) was added, and the reaction mixture was stirred overnight. The

reaction mixture was filtered over celite and concentrated *in vacuo*. The resulting material was purified by preparative HPLC (isocratic, acetonitrile/water 85/15, no acid) yielding the material in high purity (8 mg, 30%). ^{1}H NMR (400 MHz, $CDCl_3$ δ): 8.37 (s, 3H, Ar), 6.82 (d, *J* = 5.6 Hz, 3H, $C{=}ON\underline{H}CHDCH_2$), 3.74 – 3.54 (m, 51H, O-$(C\underline{H}_2)_2$-O, $C{=}ONHC\underline{H}DCH_2$), 3.43 (t, *J* = 6.8 Hz, 6H, $CH_2CH_2C\underline{H}_2O$), 3.08 (b, 3H, $CH_2CH_2O\underline{H}$), 1.60 (p, *J* = 7.2 Hz, 6H, $C\underline{H}_2CHD$-$NHC{=}O$), 1.53 (p, *J* = 7.0 Hz, 5H, $CH_2C\underline{H}_2CH_2O$), 1.45 – 1.14 (m, 48H, aliphatic). ^{13}C NMR (100 MHz, $CDCl_3$ δ): 165.83, 135.19, 128.11, 72.52, 71.53, 70.56, 70.54, 70.49, 70.47, 70.24, 69.94, 61.64, 61.64, 40.03 (t), 29.49, 29.47, 29.41, 29.40, 29.37, 29.37, 29.36, 29.18, 26.87, 25.99. FT-IR (ATR) ν (cm^{-1}): 3382 (broad), 3234, 3064, 2917, 2851, 1638, 1559, 1468, 1350, 1315, 1296, 1253, 1202, 1118, 942, 884, 723, 693.

6. Estimation of pKa values

The pKa value of ethylene glycol is 14.2 in water at 25°C[6], and of benzamide is 13 in water at 25 °C [7].

Supplementary References

1) Leenders, C. M. A. *et al.* Supramolecular polymerisation in water; elucidating the role of hydrophobic and hydrogen-bond interactions. *Soft Matter* **12**, 2887–2893 (2016).
2) Leenders, C. M. A. *et al.* Supramolecular polymerization in water harnessing both hydrophobic effects and hydrogen bond formation. *Chem. Commun.* **49**, 1963–1965 (2013).
3) Green, M. M., Andreola, C., Munoz, B., Reidy, M. P. & Zero, K. Macromolecular stereochemistry: a cooperative deuterium isotope effect leading to a large optical rotation. *J. Am. Chem. Soc.* **110**, 4063–4065 (1988).
4) Cantekin, S., Balkenende, D. W. R., Smulders, M. M. J., Palmans, A. R. A. & Meijer, E. W. The effect of isotopic substitution on the chirality of a self-assembled helix. *Nat Chem* **3**, 42–46 (2011).
5) Balkenende, D. W. R. *et al.* Enantioselective Synthesis of (R)- and (S)-1-2H-1-Octanol and Their Corresponding Amines. *Synth. Commun.* **42**, 563–573 (2012).
6) https://labs.chem.ucsb.edu/zhang/liming/pdf/pKas_of_Organic_Acids_and_Bases.pdf
7) http://sites.chem.colostate.edu/diverdi/all_courses/CRC%20reference%20data/dissociation%20constants%20of%20organic%20acids%20and%20bases.pdf
